# Polyamine Induction in Postharvest Banana Fruits in Response to NO Donor SNP Occurs via l-Arginine Mediated Pathway and Not via Competitive Diversion of S-Adenosyl-l-Methionine

**DOI:** 10.3390/antiox8090358

**Published:** 2019-09-01

**Authors:** Veeresh Lokesh, Girigowda Manjunatha, Namratha S. Hegde, Mallesham Bulle, Bijesh Puthusseri, Kapuganti Jagadis Gupta, Bhagyalakshmi Neelwarne

**Affiliations:** 1 Plant Cell Biotechnology Department, Council of Scientific and Industrial Research-Central Food Technological Research Institute, Mysore 570020, India; 2 National Institute of Plant Genome Research, Aruna Asaf Ali Marg, New Delhi 110067, India

**Keywords:** fruit ripening, ethylene, SAM decarboxylase, arginine decarboxylase, ornithine decarboxylase, Musa

## Abstract

Nitric oxide (NO) is known to antagonize ethylene by various mechanisms; one of such mechanisms is reducing ethylene levels by competitive action on S-adenosyl-L-methionine (SAM)—a common precursor for both ethylene and polyamines (PAs) biosynthesis. In order to investigate whether this mechanism of SAM pool diversion by NO occur towards PAs biosynthesis in banana, we studied the effect of NO on alterations in the levels of PAs, which in turn modulate ethylene levels during ripening. In response to NO donor sodium nitroprusside (SNP) treatment, all three major PAs viz. putrescine, spermidine and spermine were induced in control as well as ethylene pre-treated banana fruits. However, the gene expression studies in two popular banana varieties of diverse genomes, Nanjanagudu rasabale (NR; AAB genome) and Cavendish (CAV; AAA genome) revealed the downregulation of SAM decarboxylase, an intermediate gene involved in ethylene and PA pathway after the fifth day of NO donor SNP treatment, suggesting that ethylene and PA pathways do not compete for SAM. Interestingly, arginine decarboxylase belonging to arginine-mediated route of PA biosynthesis was upregulated several folds in response to the SNP treatment. These observations revealed that NO induces PAs via l-arginine-mediated route and not via diversion of SAM pool.

## 1. Introduction

The prime task of shelf life extension for tropical climacteric fruits such as banana, peach, and mango relies on the suppression of ethylene through controlled elicitation of specific metabolic regulators, which in turn counter ethylene formation. While the major biochemical transformation process in ripening climacteric fruits is well-known, research on the involvement of signal molecules is still scanty. Recently, much focus has been placed on the involvement of a signaling molecule, nitric oxide (NO) in fruit ripening. NO is a highly reactive gaseous free radical signal molecule involved in a large array of biochemical reactions, cellular processes, and development in various organisms including plants. In plants, NO plays a key role in coordinating various biochemical pathways, among which the signaling roles in modulating the hormonal metabolism and in turn improving quality attributes and nutritional compositions of fruits are very important. NO is known to intercept the ethylene biosynthesis pathway at various steps [1]. For instance, low concentrations of NO produced either endogenously or applied exogenously, exert substantial growth promoting and ethylene inhibitory effects [2]. It was demonstrated that maturation and senescence of plants recorded a significant decrease of NO emission, while the exogenous application of NO at such instances delayed senescence and maturation significantly [3]. An inhibitory effect of ethylene by NO has been found in a wide range of both climacteric and non-climacteric fruits. Among climacteric fruits, the inhibitory effect of ethylene biosynthesis by NO has been evident in banana, tomato, peach, apple and avocado [4,5,6]. Non-climacteric fruits like strawberry, sweet pepper and longan have also been studied for NO’s antagonistic effect on ethylene [3,7,8,9,10]. In addition to enhancing postharvest life, NO is known to provide additional nutritional value through enhanced ascorbate content [11]. Reactive oxygen species/reactive nitrogen species signaling also play a crucial role in controlling nitro-oxidative stress response during ripening of both climacteric and non-climacteric fruits [12].

Plants generate NO by various oxidative and reductive routes that play a key role in various processes such as plant growth, development, and resistance against various biotic and abiotic stresses [13,14]. Among the various pathways, nitrate reductase and mitochondria are other major sources of NO [15]. Apart from these two NO generating pathways, plasma membranes of root cells can generate NO via nitrite–NO reductase activity. A second category of NO producing enzymes operate via oxidative reaction. The best studied but uncharacterized pathway is the NO synthase-like (NOS or NOS-like) enzyme which has similar properties to animal NOS that uses L-arginine (Arg) as the substrate and produces NO and citrulline. This pathway operates in animals, microbes and algae. The existence of NOS-like enzymes in higher plants is still elusive, and the NOS-like activity detected in several studies could be the result of cooperation between discrete proteins which together can generate NO [16]. Hydroxylamine-mediated NO generation pathway is also mediated by an oxidative process [17]. Polyamines (PAs) mediated NO pathway is another oxidative pathway of NO production [18].

PAs are positively charged at physiological pH and hence they tend to bind strongly with negatively charged nucleic acids [19], acidic phospholipids and various types of proteins [20]. It is well known that supplementation of PAs provides anti-senescence effects in many plant species [21]. Such effects have obviously relevant in the context of fruit ripening since ripening is a senescence phenomenon [22]. In the case of tomato fruits, PAs inhibited the transcript accumulation of the wound inducible 1-aminocyclopropane-1-carboxylate synthase (ACC synthase; ACS) [23]. PAs treatments in pomegranate enhanced the shelf life as well as several quality attributes [24], establishing the relationship between ethylene and PAs, and the latter delayed the fruit ripening and senescence process [25]. The mechanism of PAs effects is linked chiefly to the reduction of ethylene by post-transcriptional modification of ethylene biosynthesis enzymes rather than the reported feedback inhibition as each of these shares a common precursor, S-adenosyl-l-methionine, SAM [26].

As stated earlier, PAs induce NO in plant systems. PAs, spermidine (Spd) and spermine (Spm) greatly induced NO release in *Arabidopsis* seedlings whereas Arg and putrescine (Put) had little or no effect [18]. Treatment of Banana fruits with PAs delayed ripening-related processes such as softening, development of peel color, ACC synthesis while suppressing the rate of ethylene production, respiration and 1-aminocyclopropane-1-carboxylate oxidase (ACC oxidase; ACO) [27]. Similarly, Spd and Spm were shown to reduce ethylene synthesis by inhibition of ACS [28]. Under anoxic conditions, NO reacts with PAs leading to generation of NONOates. Spermine NONOate has been favored as a chemical NO donor because it spontaneously releases NO in aqueous solution, and this shows a direct chemical link between PA and NO [29].

**Figure 1:**
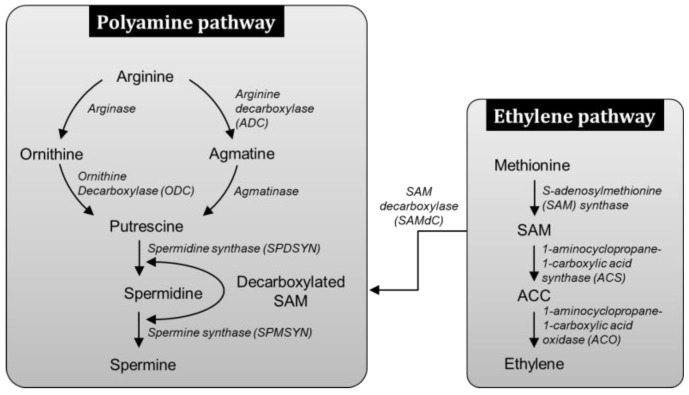
Polyamine biosynthesis pathway and its linkage to ethylene biosynthesis pathway. S-adenosylmethionine (SAM) is a precursor for ethylene biosynthesis which is synthesized from methionine by SAM synthase. The generated SAM is then converted to ethylene by the action of 1-aminocyclopropane-1-carboxylic acid synthase and 1-aminocyclopropane-1-carboxylic acid oxidase enzymes. SAM also gets decarboxylated upon SAM decarboxylase (SAMdC) action and can act as precursor for spermidine and spermine synthesis. Putrescine can be directly synthesized from ornithine by the action of ornithine decarboxylase or indirectly from arginine by arginine decarboxylase.

SAM is an immediate precursor of ethylene biosynthesis and is synthesized from methionine mediated by SAM synthase (Figure 1). The rate-limiting step in ethylene biosynthesis is the conversion of SAM to ACC by the enzyme ACS [30]. ACC is then converted to ethylene, CO_2_ and HCN by ACO. Put can be directly synthesized from ornithine (Orn) by the action of ornithine decarboxylase (ODC) enzyme, or it can be produced indirectly from Arg by arginine decarboxylase (ADC). SAM decarboxylase (SAMdc) has probably been the rate limiting enzyme that provides aminopropyl moiety used by Spd synthase (SPDSYN) and spermine synthase (SPMSYN) to convert Put to Spd and Spm respectively. SAM acts as a common precursor to both PA and ethylene pathway and thus, increase in PA biosynthesis, particularly via SAMdc activity is likely to affect the rates of ethylene biosynthesis. Moreover, much higher concentrations of PAs than those of both ACC and ethylene are indicative that changes in PAs are more likely to affect ACC and ethylene biosynthesis and vice versa [31].

Though there are reports on enhancement of polyamines by NO [32,33], the mechanism of polyamine generation via NO treatment still needs mechanistic insights. In this study we addressed whether the increase in PAs occurs via diversion of SAM pool from ethylene pathway. Therefore, PAs were measured upon SNP (NO donor) and ethylene treatment.

The experiments were conducted using Nanjanagudu rasabale (NR) fruits, a variety of India with AAB genome. However, for gene expression studies, Cavendish variety (CAV, AAA genome) (A globally popular variety of banana) was also considered to compare whether the mechanisms of PA enhancement upon NO and ethylene treatment are the same across fruits of diverse genomes.

## 2. Materials and Methods

### 2.1. Samples, Treatments, Nitrite and NO Measurements

Freshly harvested mature CAV and NR banana fruits having uniform size, shape and color were procured from local farmers. Based on our previous studies, [6] fruits were treated with NO donor 1 mM sodium nitroprusside (SNP) by dip treatment for 4 h as described previously. After treatment, fruits were placed on blotters to clear water on their surface, stored in paper envelopes at room temperature, and the sampling was done on every 5th day for a period of 20 days. Fruits treated with distilled water served as control. For HPLC analysis, ethylene in the form of ethrel (35%, *v*/*v*; Himedia) was treated to NR fruits at 2 mL L^−1^ for 4 h which served as ethylene treated control fruits, and on the subsequent day of ethylene treatment, 1 mM SNP was used for ethylene pre-treated to fruits. The effectiveness of SNP in releasing NO was checked by 3-amino,4-aminoethyl-2′,7′-difluorofluorescein diacetate (10 μM DAF-FMDA) fluorescence. Control and SNP treated banana peels were incubated wither in buffer, or 10 μM DAF-FMDA or 10 μM DAF-FMDA + 200 µM 2-4-carboxyphenyl-4,4,5,5-tetramethylimidazoline-1-oxyl-3-oxide (cPTIO) for 20 min in dark and fluorescence imaging was performed by inverted confocal laser scanning microscope and images were acquired by using LAS AF software (Leica Microsystems, Wetzlar, Germany).

### 2.2. Quality and Shelf Life

Change in peel color of fruits was measured (Minolta CR-200 colorimeter, Konica Minolta Sensing Americas, Inc., Ramsey, NJ, USA) following the procedure of McGuire [34] with the color space of L* a* b* indicating lightness, red/green value and blue/yellow color respectively. The change in the color was measured in terms of lightness, chroma and hue values. Firmness was measured using the universal texture measuring system (UTM Lloyds, LR-5K, Lloyd Instruments Ltd., Fareham, UK). A cylindrical penetration probe with a diameter of 2 mm penetrated the fruit at constant speed to a depth of 15 mm with load cell of 5 kg. The maximum force applied to break up the peel represented the peel hardness, which was expressed as “Newtons” (N) as described by Breene [35].

### 2.3. Ethylene Measurement by Gas Chromatography

Ethylene measurement was performed according to Pathak et al. [36] with few modifications. For the ethylene analysis, an individual whole banana fruit was incubated in an airtight glass jar for 1 h and 1 mL of air sample from headspace was withdrawn using a gastight Hamilton syringe and injected into a gas chromatograph (GC2010plus, Shimadzu, Kyoto, Japan). A flame ionization detector and a poly(dimethyl)siloxane capillary column (30 m × 0.32 mm ID × 0.25 mm film thickness) (Rtx-1, Restek Inc., San Diego, CA, USA) were employed for ethylene estimation. Injector and detector temperatures were 250 °C and an isothermal program was run at 30 °C. The temperature of the column and detector was set at 80 °C and 150 °C, respectively. The carrier gas N_2_ flow rate was 40 mL min^−1^; and the hydrogen pressure was 0.6 kg cm^−2^. The rate of ethylene production was expressed as µL C_2_H_4_ h^−1^ g^−1^ FW.

### 2.4. Extraction and Estimation of Polyamines

The extraction of PAs, benzoylation of authentic standards of Put, Spd and Spm (Sigma-Aldrich, St. Louis, MO, USA) as well as HPLC analysis were conducted according to the method of Flores and Galston [37]. For PAs extraction, 1 g of banana fruit pulp, and peel tissues were extracted in 10 mL of 5% cold perchloric acid and incubated on ice for 1 h. The samples were then centrifuged at 10,000× *g* for 20 min at 4 °C. The supernatant phase containing the PA fraction was stored at −20 °C overnight. For benzoylation, to the 250–500 µL of perchloric acid extract 1 mL of 2 N NaOH was added followed by 10 µL of benzoyl chloride. The samples were vortexed for 10 s and incubated at room temperature for 20 min. Then, 2 mL each of saturated NaCl and diethyl ether were added, mixed and centrifuged at 5000× *g* for 5 min at 4 °C. 1 mL of ether phase was collected from all the samples and was kept for evaporation in warm water. Once the ether phase was evaporated completely, the samples were re-dissolved in 100 µL of methanol.

Benzoylated PAs and standards were estimated using a HPLC system (LC-20A; Shimadzu, Kyoto, Japan) equipped with a dual pump and UV-Visible detector and chromatograms were acquired at 254 nm. The elution system consisted of MeOH/H_2_O (64:36) solvent in isocratic mode with a flow rate of 1 mL min^−1^. The benzoylated PAs were eluted through a C_18_ column (300 × 4.6 mm i.d. with pore size of 5 μm). A relative calibration procedure was used to determine the PAs in the samples using Put, Spd and Spm standards. Peak areas and retention times were measured by comparing with standard PAs. Results were expressed as nanomoles per gram fresh weight (mean ± SE).

### 2.5. RNA Extraction, DNase Treatment and RNA Quantification

RNA extraction was carried out for control and SNP (1 mM) treated CAV and NR fruits. RNA extraction was carried out using the method of Asif et al. [38]. Contaminating traces of DNA was removed from the RNA preparations by RNase free DNase I treatment. After purification, the RNA was quantified using Nanodrop 1000 (Thermo Scientific, Waltham, MA, USA) and purity was assessed by the ratio of A_260_/A_280_.

### 2.6. First Strand cDNA Synthesis

The first strand cDNA synthesis was performed for RNA samples extracted on every 5th day after treatment of fruits (0, 5, 10, 15 and 20th day) using Verso cDNA synthesis kit (Thermo Scientific, Waltham, MA, USA). 2 µg of total RNA was reverse transcribed according to Manufacturer’s protocol.

### 2.7. Primer Designing

Sequences of five PA pathway genes (*ADC*, *Arginase*, *SAMdc*, *SPMSYN* and *SPDSYN*) and two ethylene pathway genes (*ACS* and *ACO*) were retrieved from either NCBI nucleotide database or banana genome database and the PCR primers were designed using Oligo Explorer software and were crosschecked with Oligo Analyzer online tool of Integrated DNA Technologies (http://eu.idtdna.com/calc/analyzer). *GAPDH* gene was used as reference gene for expression studies. All the primers used in the study are listed in Table S1.

### 2.8. Semi-Quantitative RT-PCR

PCR amplifications were performed using PCR mixture (20 μL) that contained 2 μL of tenfold diluted cDNA as template, 1X PCR buffer, 200 μM dNTP mix, 1 U of Taq DNA polymerase, and 0.2 μM of each gene specific primer. PCR was performed at initial denaturation at 94 °C for 4 min, 30 cycles (94 °C for 30 s; 55–60 °C for 45 s; 72 °C for 1 min), and final elongation (72 °C for 5 min) using a thermal cycler. The PCR products obtained were separated on 1.8% agarose gel, stained with ethidium bromide (0.001%), and documented in a gel documentation system (Herolab GmbH Laborgerate, Wiesloch, Germany).

### 2.9. Quantitative Real Time PCR

The PCR reactions were set up in 20 µL reaction volume containing 10 µL of 2X Maxima SYBR Green Master Mix (Applied Biosystems, Foster city, CA, USA), 0.5 µL of each gene specific forward and reverse primers, 2 µL of tenfold diluted cDNA, and the volume was made up to 20 µL with nuclease free water. No template control was kept for each primer. Expression levels were analyzed for all the treated samples in duplicates in a single 96-well reaction plate, and the reproducibility of qRT-PCR results was confirmed in two independent experiments. The qRT-PCR experiments were done using an ABI PRISM 7700 Sequence Detection System (Applied Biosystems, USA). The PCR program included initial activation step of 2 min at 50 °C, 10 min at 95 °C followed by 40 successive cycles of 15 s denaturation at 95 °C and 1 min annealing and extension at 60 °C. Subsequent to PCR amplification, melt curve analysis of the amplicons was performed to check the specificity of the primers using Dissociation Curve Analysis Version 1.0 (Applied Biosystems, USA). Relative gene expression was calculated according to 2^−ΔΔCT^ method [39]. The expression data was normalized against the GAPDH reference gene, and the fold change in expression was relative to the expression of control fruit at that corresponding stage, which was set to 1.

### 2.10. Statistical Analysis

Experiments of fruit texture and color measurement estimations were done twice with six replicates for each analysis. Data were subjected to one-way analysis of variance (ANOVA) followed by post hoc Duncan’s Multiple Range Test using SPSS 17 (SPSS Inc., Chicago, IL, USA) for determining significant differences. Ethylene measurements were done twice with three replications, and the data was subjected to one way ANOVA followed by post hoc least significant difference (LSD). A difference was considered significant when *p* < 0.05. HPLC analysis was done twice with two replicates, and the data was subjected to one-way ANOVA followed by Tukey honestly significance difference (HSD) test with the significances at *p* ≥ 0.001.

## 3. Results

### 3.1. NO Treatment Differentially Affects the Shelf Life

In control fruits, the lightness as indicated by the L* values, gradually started decreasing after the 10th day, and on the 20th day the least lightness value had reached 62.54, Table S2. SNP treated banana fruits retained the lightness till the 10th day. Even on the 15th day, only a marginal reduction in lightness was detected, and by the 20th day, the fruits were over-ripened and resulted in lower lightness values due to the development of dark patches. The chroma values were not significantly affected during the later stages of ripening though on the 5th day after treatment; lower chroma values were observed in SNP treated fruits. Both treated and control fruits showed positive hue values on the 20th day. The difference was insignificant during the initial period indicating that SNP did not impart a significant effect on peel color retention in banana fruits.

**Figure 2:**
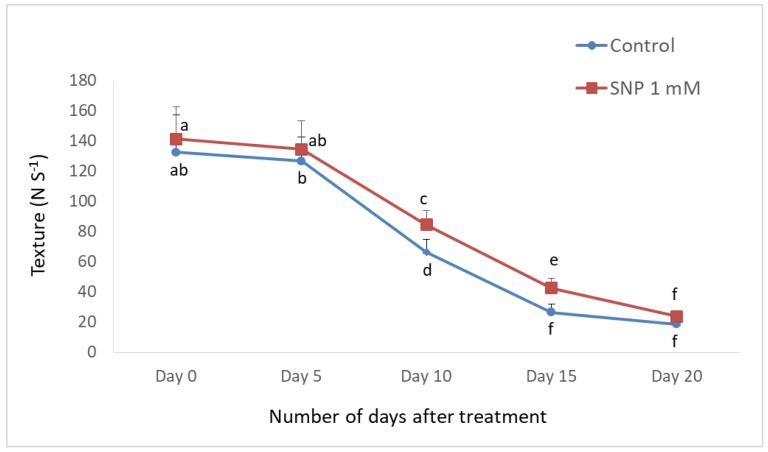
Effect of sodium nitroprusside on texture of banana fruit. Measurements were taken once in every 5 days.

**Figure 3:**
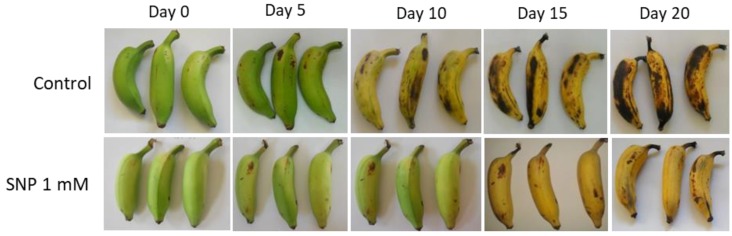
Representative images of banana fruit phenotypes in control and in response to sodium nitroprusside treatment.

Treated fruits were subjected to axial and radial punctures, and the maximum force required to rupture the fruit was recorded as Newtons (relative firmness) and presented in Figure 2. There was a steady loss of firmness during the progression of ripening until the post-climacteric stage in both the control and SNP treated fruits. SNP treated fruits retained relatively higher firmness even on the 20th day and could delay the fruit softening as measured by texture firmness. The firmness of fruits at the initial stages was significantly higher in treated fruits with 134.64, 84.52 and 42.52 N s^−1^ on day 5, 10 and 15 respectively when compared to control fruits with 126.62, 66.54 and 26.54 N s^−1^ on the corresponding days (Figure 3).

### 3.2. SNP Treatment Leads to Reduced Ethylene Production

**Figure 4:**
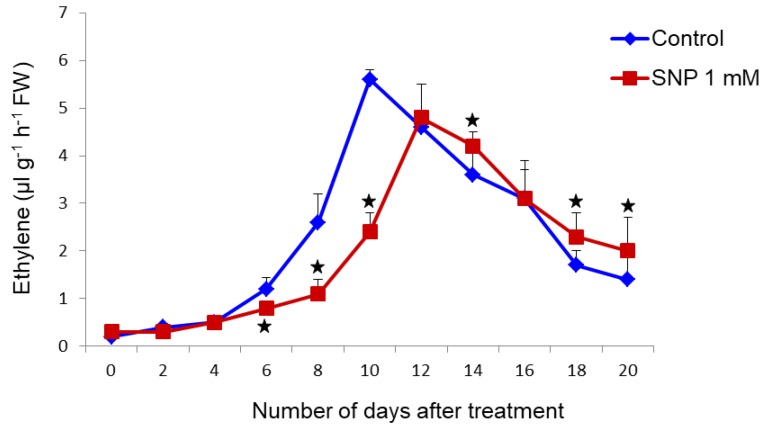
Changes in ethylene levels in treated banana fruits measured by gas chromatography. The values are means of three replicates. *Mean difference is significant at the *p* ≥ 0.05 level as analyzed by one-way analysis of variance followed by post hoc test least significance difference.

Control and treated fruits recorded low ethylene levels in the pre-climacteric stage followed with a characteristic ethylene peak at their climacteric. SNP treatment significantly delayed the ethylene burst and delayed the ethylene peak till the 12th day. In control fruits, the peak was observed on the 10th day (Figure 4). Interestingly, the ethylene level in the SNP treated fruits on the peak day (day 12) was lower than that of control fruits on their corresponding peak day (day 10).

### 3.3. Acceleration of Putrescine (Put), Spermidine (Spd) and Spermine (Spm) Production in Response to SNP Treatment

Two types of controls were employed in the study, viz., untreated control fruits and ethylene treated control fruits. Levels of PAs were significantly increased in the ethylene treated control fruits compared to the untreated control fruits both in pulp and peel of banana fruits (Figure S1).

**Figure 5:**
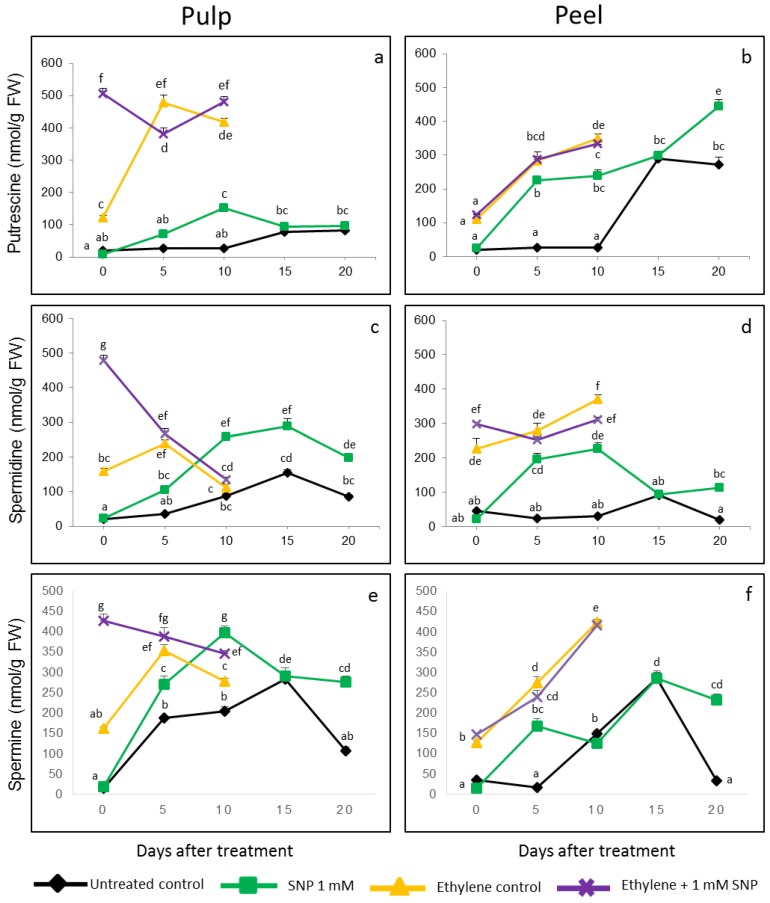
Effect of sodium nitroprusside treatment on polyamine levels in untreated and ethylene pre-treated banana fruits. (**a**) Putrescine levels in pulp of fruits; (**b**) Putrescine levels in peel of fruits; (**c**) Spermidine levels in pulp of fruits; (**d**) Spermidine levels in peel of fruits; (**e**) Spermine levels in the pulp of fruits; (**f**) Spermine levels in the peel of fruits. The data was subjected to one-way analysis of variance followed by post hoc test Tukey honestly significance difference (HSD) with the significances at *p* ≥ 0.001, and values with same superscript were found not significantly different from each other.

Put level in pulp of untreated control fruits increased from the day of treatment till the 20th day accounting for 82.14 nM g^−1^ FW. However, the ethylene treated control fruits recorded higher Put levels every day, the highest being on the 5th day with 478.23 nM g^−1^ FW. The SNP treatment for both untreated and ethylene treated fruits showed increase in the levels of Put. Untreated control fruits recorded a maximum increase of Put by 5.8 folds on the 10th day upon treating with 1 mM SNP. Ethylene treated fruits recorded a maximum increase of 4.19 folds on the 0th day and insignificant changes on the subsequent days when treated with 1 mM SNP Figure 5a. The Put levels in the peel of untreated control fruits and ethylene treated control fruits were also found to increase with the progression of ripening. The highest Put level in the peel of untreated control fruits was on the 15th day with 289.18 nM g^−1^ FW, and in the ethylene treated control fruits, the highest Put level in the peel was 348.53 nM g^−1^ FW on the 10th day after treatment Figure 5b. Unlike pulp, the Put level in peel steadily increased till the 20th day after SNP treatment while declining in pulp after the 10th day.

Spd level in pulp of untreated control fruits increased steadily till the last day of the observation. The maximum Spd content was observed on the 15th day with 155.45 nM g^−1^ FW, and the level of Spd was found increased by 1 mM SNP treatment on all the stages, and the maximum increase was found on 5th day and 10th day with 2.95 folds. In ethylene treated control fruits, the highest Spd level was recorded on the 5th day with 238.37 nM g^−1^ FW in the pulp, which decreased on the 10th day. SNP treatment to the ethylene treated fruits, increased the Spd levels in the pulp 2.99 folds on the 0th day; however, the increase on the subsequent days was insignificant (Figure 5c). Maximum Spd levels in the peel of untreated control fruits was recorded on the 15th day with 90.55 nM g^−1^ FW, and the highest increase was observed on the 10th day, 7.6 folds compared to the untreated control. The peel of ethylene treated control fruits showed an increasing pattern of Spd levels with 370.62 nM g^−1^ FW on the 10th day. SNP treatment increased the Spd levels on the 0th day, but an insignificant decrease was found on the 5th and 10th day Figure 5d.

Spm levels in the pulp of untreated control fruits increased till the 15th day with a subsequent decrease on the 20th day. The highest Spm content of 283.23 nM g^−1^ FW was recorded on the 15th day. The SNP treated fruits showed consistent increase of Spm levels with a maximum enhancement of 1.93 folds on the 10th day. However, in ethylene treated control fruits, the Spm levels were found decreased on the 10th day and the maximum level was recorded on the 5th day with 353.68 nM g^−1^ FW. SNP treatment to ethylene treated fruits marginally increased the Spm levels on the 5th and 10th day with maximum increase of Spm by 2.63 folds on the 0th day (Figure 5e). The Spm levels in the peel of untreated control fruits also showed an increasing trend till the 15th day with 285.64 nM/ g FW. The SNP treatment upregulated the Spm levels on the 5th day by 10.24 folds and 7.10 folds on the 20th day Figure 5f. The Spm level was either not much affected or insignificantly decreased on the other stages of the observation. In the peel of ethylene treated control fruits, the Spm levels increased upon the progression of ripening with a highest Spm level of 424.36 nM g^−1^ FW on the 10th day. The SNP treatment to ethylene treated fruits did not show much variation in the peel of the fruits.

### 3.4. SNP Down-Regulates Genes Involved in Ethylene Biosynthesis

**Figure 6:**
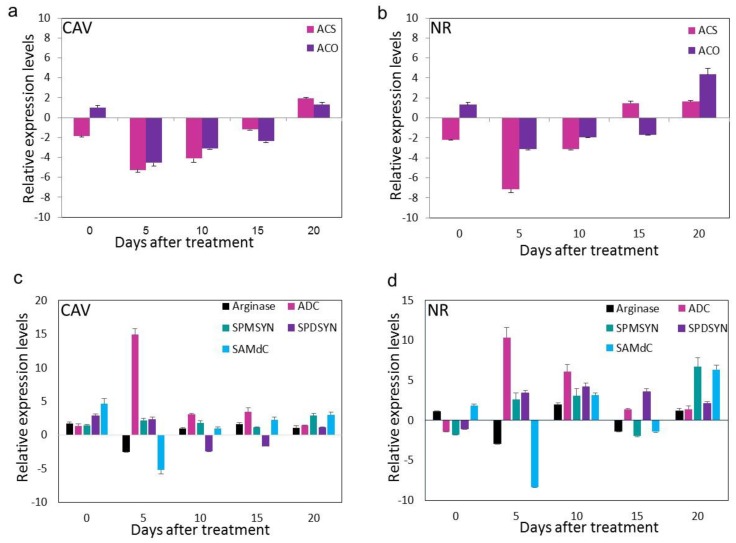
Relative expression of ethylene pathway [1-aminocyclopropane-1-carboxylic acid synthase (*ACS*) and 1-aminocyclopropane-1-carboxylic acid oxidase (*ACO*)] and polyamine pathway (Arginase, Arginine decarboxylase (*ADC*), Spermidine synthase (*SPDSYN*), Spermine synthase (*SPMSYN*), and S-adenosylmethionine decarboxylase (*SAMdC*) as determined by qRT-PCR in sodium nitroprusside (SNP) treated fruits of Nanjanagudu rasabale (NR, AAB genome) and Cavendish (CAV, AAA genome) banana fruits in different days after treatment with SNP. (**a**) Effect of SNP on expression of ethylene pathway genes, *ACS* and *ACO* in CAV fruit pulp; (**b**) Effect of SNP on expression of ethylene pathway genes, *ACS* and *ACO* in NR fruit pulp; (**c**) Effect of SNP on expression of polyamine pathway genes, *SAMdC*, *Arginase*, *SPDSYN*, *SPMSYN*, and *ADC* in CAV fruit pulp; (**d**) Effect of SNP on expression of polyamine pathway genes, *SAMdC*, *Arginase*, *SPDSYN*, *SPMSYN*, and *ADC* in NR fruit pulp.

*ACS* and *ACO* were downregulated up on treatment with SNP. *ACS* was downregulated by 5.26 and 4.1 folds on the 5th and 10th day after treatment in CAV banana and 7.14 and 3.12 folds in NR banana fruit on the same days Figure 6a,b. Expression of *ACO* was also decreased by 4.5, 3.1 and 2.3 folds on 5th, 10th and 15th days in CAV and 3.12, 1.92 and 1.7 folds on the same days in NR fruits. Similar result was observed in semi-quantitative RT-PCR experiments suggesting that SNP down-regulates genes involved in ethylene biosynthesis (Figure S2).

### 3.5. SNP Increases PA Biosynthetic Genes via Arg-Mediated Route and Not by Precursor SAM Pool Diversion

In PA biosynthesis pathway, totally five genes were studied for their expression pattern in response to SNP treatment. In CAV, *Arginase* was upregulated at the initial stages of ripening slightly but decreased on the 5th day by 2.5 folds (Figure 6c). After the 5th day, there was not much change in the expression levels, except 1.65-fold increase on the 15th day while the change was not very significant. In NR fruits, the expression levels were found increased by 1.08, 1.95 and 1.17 folds on 0th, 10th and 20th day, but an intermittent downregulation was observed on 5th and 15th day by 2.9 and 1.35 folds (Figure 6d). Interestingly, the most upregulated gene among all the PA pathway genes was *ADC* where a 14.93 and 10.39 fold increase was observed on the 5th day in CAV and NR fruits respectively. In CAV, the upregulation was persistent in all the subsequent stages. In NR, 6.1 folds upregulation was observed on the 10th day and there were not many differences on the later stages. *SAMdc* was found significantly upregulated by SNP treatment on all the stages of ripening except on the 5th day where 5.14-fold downregulation was observed compared to control fruits. The upregulation on 0th, 10th, 15th and 20th days was 4.62, 1.01, 2.27 and 3.02 folds respectively. Still, the upregulation was not very consistent in NR. However, the upregulation was obvious intermittently on the 0th, 10th and 20th day by 1.84, 3.14 and 6.36 folds respectively. The increase in the expression of *SPMSYN* was apparent in both CAV and NR, though not very prominently. Increase by 2.2 and 2.87 folds was recorded on 5th and 20th day in CAV and 2.62, 3.05- and 6.69-fold increase was observed on the 5th, 10th and 20th day in NR fruits. Similarly, 2.89- and 2.32-fold upregulation of *SPDSYN* was observed on the 0th day and 5th day in CAV and a more apparent increase of 3.46, 4.22, 3.62 and 2.12 folds on 5th, 10th, 15th and 20th day was observed in NR fruits Figure 6c,d.

## 4. Discussion

The delaying effects of NO on fruit ripening by its inhibitory effect on the major ripening hormone ethylene are well studied in various fruits as well as in banana [5,6]. In our study, banana whole fruits were treated with the most common NO donor, SNP for analyzing the effects of NO. This treatment was effective as it generated NO and nitrite in peels (Figure S3) Though this treatment is also known to generate cyanide, SNP is a well-accepted NO donor used for studying effects of NO on postharvest shelf life, particularly owing to its efficiency in endogenous NO generation, cost-effectiveness and simple usage [40,41]. Moreover, it is known that the lateral effects of cyanide toxicity could be alleviated by the in-built detoxification system of higher plants which acts primarily through a key enzyme β-cyanoalanine synthase (β-CAS). It was observed that two homologous genes, *MdCAS1* and *MdCAS2*, encoding Fuji apple β-CAS homologs function in the detoxification of cyanide, a co-product of ACO-catalyzed ethylene synthesis. Further, these genes were found upregulated with exogenous ethylene treatment and mechanical wounding suggesting that the cyanide generated through any external application would also be detoxified by β-CAS [42] NO has been attributed to downregulate several cell wall degrading enzymes conferring higher fruit firmness. Cheng et al. [5] reported that SNP treated banana slices recorded lower activities of polygalacturonase, pectin methyl esterase and endo-β-1,4-glucanases and maintained higher contents of acid-soluble pectins and starch which resulted in reduced pulp softening. In addition, in our other study, NO downregulated major cell wall hydrolysis genes such as polygalacturonase, pectate lyase, pectin methyl esterase, β-galacturonase and expansins thus retaining higher texture in SNP treated fruits [unpublished data]. Similarly, in peach fruits NO treatment retarded the loss of firmness by maintaining cell membrane integrity and reduced electrolyte leakage [43]. The effect of SNP on color retention was significant only at climacteric stage, and it was insignificant at initial and later stages of ripening as evident by the lightness, chroma and hue values. In papaya fruits, NO treatment effectively caused delay of fruit ripening by suppressing ethylene and enhanced firmness [44]. Since it is known that NO delays ripening primarily by its antagonizing ethylene, lower ethylene levels were observed which resulted in delayed climacteric peak (by 2 days) in the SNP treated fruits, which also coincided with lower ethylene level. Direct effect of NO on ethylene levels is reported in several studies where NO was used as a tool for postharvest management of fruit ripening.

PA mediated ethylene interception is an indirect mechanism where PAs are believed to crosstalk with unidentified mechanism to reduce ethylene by inhibiting ACS and also by competitive action on SAM, a common precursor for both ethylene and PA biosynthesis. In the present study, the effects of SNP on PAs and ethylene metabolism and associated gene expression was investigated in ripening banana fruit, to determine whether its effect is via PAs or directly on ethylene.

Three major PAs, Put, Spd and Spm were enhanced at various stages after treatment. While all the three PAs were recorded in significant levels in the present study, Adão et al. [45] observed that Spd and Put were the major PAs in ‘Prata’ banana fruits and the levels of Spm were below the quantification limit of the analytical technique. Contrastingly, Spm levels were predominant among all the PAs at few stages in the current study. It was interesting to note that ethylene treatment strikingly increased the PA content of fruits. The enhanced PA levels upon exogenous ethylene treatment are not in accordance to the general belief of inverse relationship between ethylene and PA owing to the competition for common precursor SAM. The hypothesis of inverse relationship between these two major biosynthesis pathways sharing a common precursor is supported with many confirmatory studies in wide range of plants [46,47]. However, there are numerous reports where ethylene-enhanced PA levels also have been observed. For instance ethylene treatment to the four-day cultured cells of tobacco increased PAs Spd and Spm levels [48]. Moreover, transgenic tomato plants with overexpressed *SAMdc*, apart from enhanced PAs, also recorded increased ethylene levels compared to non-transgenic control fruits suggesting that both ethylene and PA pathway can co-exist and function in harmony instead of mutual inhibition [49]. Higher concentration of aminoethoxyvinylglycine, an ethylene inhibitor, slightly decreased mesocarp PA levels during peach fruit ripening indicating the non-existence of the inverse relation of ethylene and PA biosynthesis [50]. The enhanced SAM pool by exogenous ethylene treatment may also be utilized by the PA biosynthesis pathway influencing increase in both ethylene and PA levels. In leaf discs of rape plant, it was observed that though Spd increased and Put decreased moderately upon ethylene inhibitor treatment in control tissues, the Put and other PAs declined upon ethylene inhibitor treatment in stressed tissues indicating that the fluxes of SAM towards either PA or ethylene are extremely responsive to stress and environmental challenges [51].

SNP significantly enhanced the PA levels in non-ethylene treated control fruits. It was interesting to note that the increase in the PA levels in untreated control fruits post SNP treatment correlated with the progression of ripening and the peak in PA accumulation was observed during climacteric and post-climacteric stages of ripening. These results also support the argument that PA and ethylene pathways need not be mutually inhibiting and can co-exist. Long term and higher dose of SNP treatment enhanced accumulation of intracellular Put and Arg in leaves of *Medicago truncatula* plants which was correlated to the maximum NO content of the leaves and this was believed to occur as part of the plant defense against nitrosative stress caused by SNP [52]. It was also observed that PA levels did not rise upon the 1-methylcyclopropane treatment of tomato fruits, but started increasing when the fruit ripening resumed indicating that PAs influence the rate of ripening/ over-ripening by counteracting the ethylene effects of ripening rather than directly regulating ripening [53]. In tomato, it was observed that though 1-methylcyclopropane treatment has inhibited autocatalytic ethylene production, but did not affect the overall SAM levels and the expression of *SAMdc* was upregulated by ethylene treatment indicating that unlike banana, tomato fruits consume SAM simultaneously during ripening to ensure a high rate of ethylene and PA production along with other SAM dependent physiological processes [54]. Kushad et al. [55] also stated that the ethylene and PA biosynthetic pathways are not actively competing for the same substrates at any given stage of the avocado fruit development and ripening since 5’-methylthioadenosine molecules produced during the PA and ethylene biosynthesis were actively metabolized to 5-methylthioribose and 5-methylthioribose-1-phosphate, the two intermediates involved in the regeneration of SAM.

Catabolic pathway of PAs comprised of Diamine oxidases (CuAO) and Polyamine oxidases (PAO). Several recent studies in both climacteric and non-climacteric fruits revealed the role of these oxidases in maintaining homeostasis of PA levels in cells [56,57,58]. Previously, it was demonstrated that the decrease in PAs levels during ripening in grapes was accompanied by upregulation *CuAO* and *PAO* genes and concomitant increase in their enzymatic activity and H_2_O_2_ levels [55]. However, in our studies, PAs content was low at the initial stages of ripening followed by an increase during post-climacteric stage in control fruits suggesting higher PA catabolic gene expression during initial stages of ripening. The sharp increase in the accumulation of PAs upon SNP and ethylene treatment suggests a possible down-regulation of PA catabolic genes. The increase of PAs by SNP treatment also corroborated at genetic level in terms of the upregulation of PA biosynthesis pathway genes and downregulation of ethylene pathway genes. In plants, polyamine mediated NO is synthesized by two major routes, one is directly from Orn through the activity of ODC, and the other from Arg by the action of two enzymes ADC and Agmatinase. However, in certain plants, the ODC mediated NO synthesis is either completely absent or present in less active state. In *Arabidopsis thaliana*, the ODC enzyme is absent and PA synthesis occurs via ADC mediated pathway [59]. Further, in *M. truncatula* plants, though ODC activity was detected in lower levels, the gene coding for the eukaryotic-type ODC in the genome sequence could not be identified [50]. Similarly, in our study, several attempts to amplify *ODC* gene was not successful, with an additional hurdle of non-availability of gene sequence data in genome of banana [60].

Increase in the ADC activity and Put concentration in sliced banana fruit was correlated with the increase in the ethylene evolution. Put accumulation was largely suppressed by ADC inhibitor and ODC inhibitor had no effect on Put accumulation. Further, SAM and ACS inhibitors also suppressed the ethylene and Put levels suggesting that ethylene produced in the wounded pulp induced the ADC activity and Put accumulation and in banana, Put is mainly synthesized through the Arg-mediated pathway [61].

The gene expression studies were carried out in two varieties of banana, to check whether the effects of NO are similar in different varieties of banana with varying genome content. The expression studies clearly indicated that ethylene pathway genes *ACS* and *ACO* were downregulated by SNP treatment in both the varieties. The delay in ripening-associated changes in SNP treated banana was attributed primarily to transcriptional downregulation of *ACO* gene as also observed in an earlier study [5]. The expression studies of PA pathway genes revealed that *SAMdc* was not much affected by the SNP treatment. *SAMdc* is the enzyme responsible for decarboxylation of SAM to synthesize decarboxy-SAM which in turn acts as precursors for SPMSYN and SPDSYN to form Spm and Spd respectively. The increase in PA accumulation by SNP treatment through diversion of SAM pool towards the PA biosynthesis would have upregulated the *SAMdc* expression as this enzyme is an intermediate between ethylene and PA pathway. However, in NR and CAV fruits, the expression of *SAMdc* was downregulated significantly indicating that the increase in PA levels was not through SAM diversion. Interestingly, the expression of *ADC* was significantly upregulated in both NR and CAV fruits from 5th day till the 20th day indicating that the Put accumulation upon SNP treatment mainly occurs through Arg-mediated PA biosynthesis. In banana, it is reported that PAs are biosynthesized majorly through Arg-mediated pathway and the existence of Orn mediated pathway is doubtful owing to the negligible ODC activity and non-availability of *ODC* gene sequence in the banana genome. The increase in the expression of *SPMSYN* and *SPDSYN* in NR fruits was observed in all the stages after 5th day and this increased expression may be attributed to the conversion of Put synthesized through Arg-mediated route by *SPMSYN* and *SPDSYN* enzymes and not necessarily from decarboxy-SAM synthesized by the action of *SAMdc*. Similar expression pattern was observed for *SPMSYN* and *SPDSYN* in CAV except that *SPDSYN* showed a downregulation at few stages of ripening. Arginase, responsible for the conversion of Arg to Orn was found not much affected by SNP treatment in both the varieties. However, it showed downregulation on 5th day in both the fruits which may be explained as the conversion of Arg to Orn was downregulated and more of Arg was diverted towards agmatine by upregulation of *ADC*. As PAs are also biosynthesized from amino acid Arg via arginase and *ADC*; and NO, via NOS-like activity, the availability of this particular amino acid might also influence PA-NO metabolism in plants and other organisms [62].

## 5. Conclusions

In summary, the external application of NO in the form of SNP triggers the levels of PAs via Arg-mediated route and not via competitive diversion of common SAM precursor. Down-regulation of *SAMdc* gene at climacteric stage of ripening supports this hypothesis, while unravelling that the PA biosynthesis and ethylene pathway complement each other (not mutually inhibiting), which is evident from the prominent increase of PA levels in ethylene treated fruits. The study also reiterates the findings of Yoza et al. [63] that PA biosynthesis in banana occurs majorly through Arg-mediated route. The preliminary information brought out on the presence of ODC gene, although could not be amplified even after repeated attempts in banana, paves way for more detailed studies in future.

## Supplementary Materials

The following are available online at https://www.mdpi.com/2076-3921/8/9/358/s1, Figure S1. Representative chromatograms of polyamine HPLC profiles of standard mix and treated banana pulp extracts. Figure S2. Relative expression of ethylene pathway (*ACS* and *ACO*) and polyamine pathway. Figure S3: Nitric oxide DAF-FMDA fluorescence in Banana peels in response to SNAP treatment. Table S1. Oligo nucleotide primers used for expression studies of polyamine pathway and ethylene pathway genes. Table S2. Effect of SNP treatment on color parameters during banana fruit ripening.
