# Supplementary material for: Polyamine Induction in Postharvest Banana Fruits in Response to NO Donor SNP Occurs via l-Arginine Mediated Pathway and Not via Competitive Diversion of S-Adenosyl-l-Methionine
Source: Antioxidants (Basel). 2019 Sep 1;8(9):358. doi: 10.3390/antiox8090358 (PMC6769871; doi:10.3390/antiox8090358)
Supplement: Supplementary file 1 [file antioxidants-08-00358-s001.pdf]

Supplementary Materials:

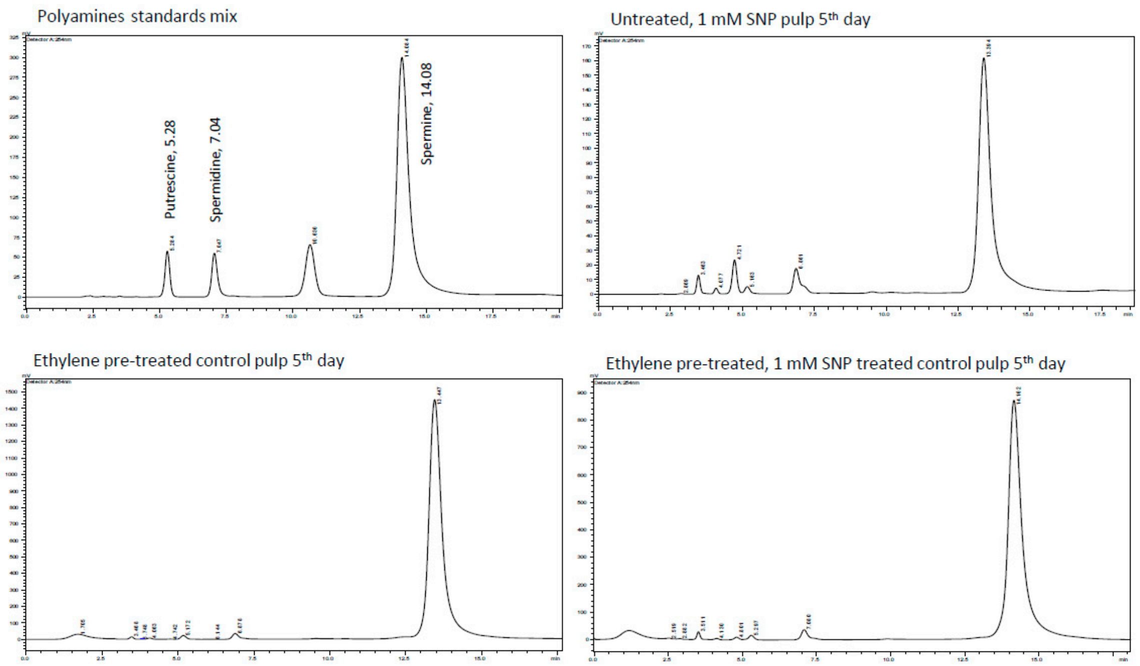

Figure S1. Representative chromatograms of polyamine HPLC profiles of standard mix and treated banana pulp extracts. The peaks for putrescine, spermidine and spermine were observed at 5.28, 7.04 and 14.08 minutes respectively in standard mix in an isocratic mode of elution consisting of MeOH/H_2O (64:36). Standards were of 2.5 mg mL^{-1} concentration and 5 µL each of standard was mixed and used for analysis.

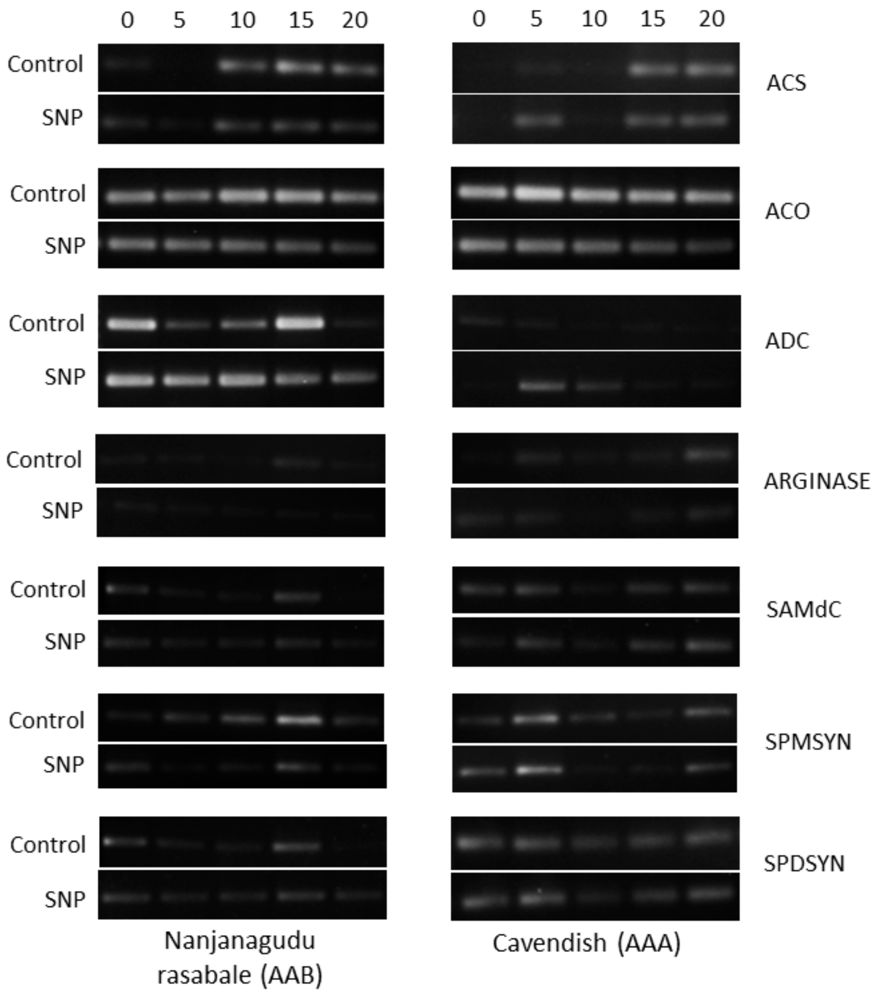

Figure S2. Relative expression of ethylene pathway (*ACS* and *ACO*) and polyamine pathway (*Arginase*, *ADC*, *SPDSYN*, *SPMSYN*, and *SAMdc*) as determined by semi-quantitative RT-PCR in SNP treated fruits of Nanjanagudu rasabale (NR, AAB genome) and Cavendish (CAV, AAA genome) banana fruits.

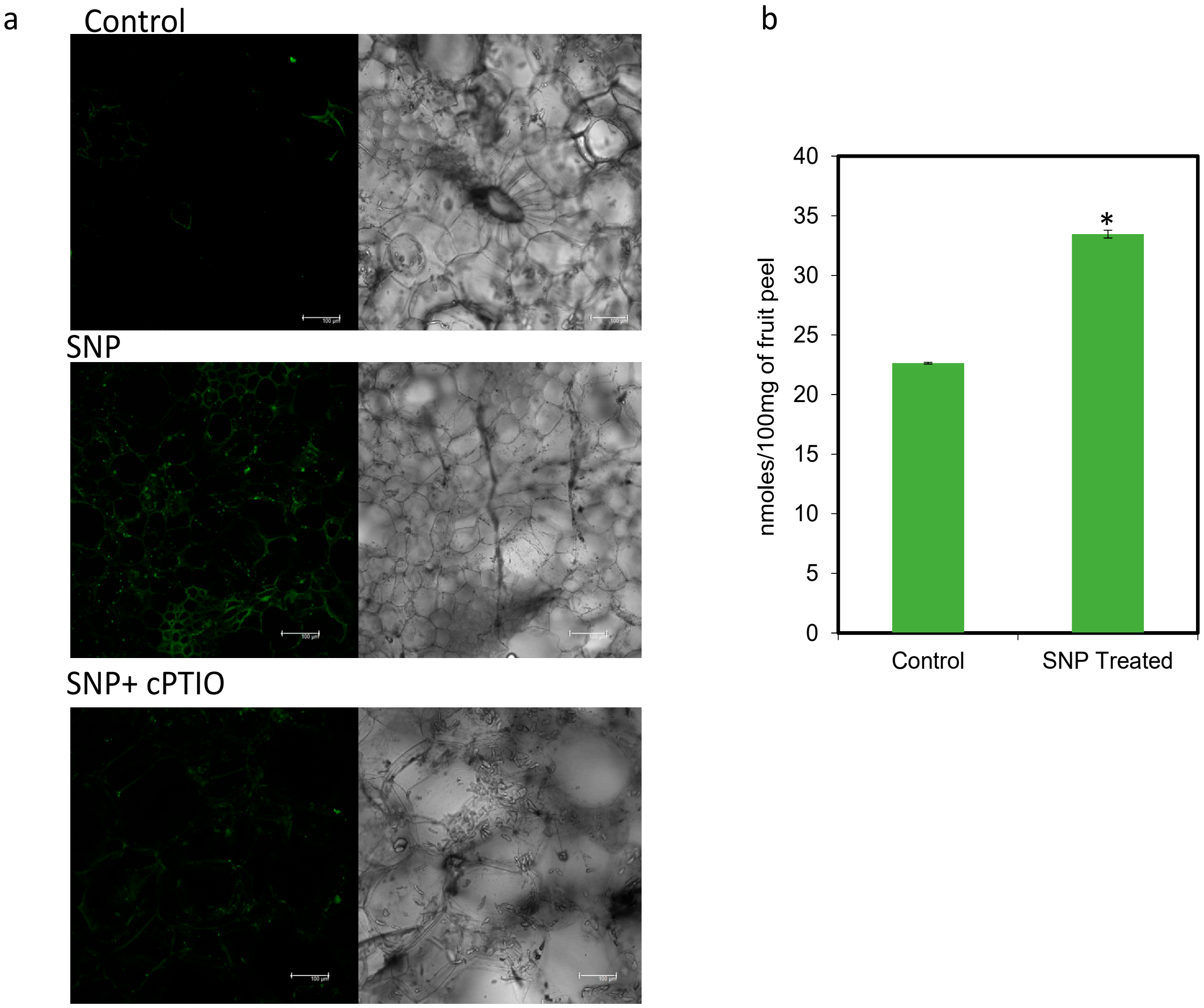

Figure S3. Nitric oxide DAF-FMDA fluorescence in Banana peels in response to SNP treatment. 200 µM cPTIO was used as control. Image is a representative of 3 independent replicates. B. Nitrite levels in response to SNP treatment. T test was performed and significant difference was $p < 0.005$.

Table S1. Oligonucleotide primers used for expression studies of polyamine pathway and ethylene pathway genes.

Sl. No.	Gene	Primer name and sequence	Amplicon (bp)	Accession No.
		Polyamine pathway genes		
1	S-adenosyl methionine decarboxylase (SAMdC)	SAMdCF – CAACCATCTCCACATCGTAG<br>SAMdCR1 - TGCCCTCTCAGTATTCAGC	207	XM_009411923.2
2	Spermine synthase (SPMSYN)	SPMSYN261F1 – GTGCCATGAGACGGTGGTGTC<br>SPMSYNR - GCAATCATCTCTTGGTAGGC	257	XM_018830840.1
3	Spermidine synthase (SPDSYN)	SPDF1 – GGAGGCGGTGATGGTGGTGT<br>SPDR1 - GTCCAAGCATAAYTCACAGAG	413	XM_009414039
4	Arginase	ARGNS158F – GACCACTCCATATCATTTCCAG<br>ARGNS158R - CCTCCCTCCATTATTCGTGC	158	XM_009402527
5	Arginine decarboxylase (ADC)	ADC296F – GTGGAGGACATCATGGAGTT<br>ACD296R – TGCTTGGTGCGGAGCTTGGC	296	XM_009420048
		Ethylene pathway genes		
6	1-aminocyclopropane carboxylate synthase (ACS)	NRACS-140F-CGTCAACGAGAAAAGGATG<br>NRACS-140R-TGTAGGCGATGTGAATCAG	140	AF056163.1
7	1-aminocyclopropane carboxylate oxidase (ACO)	NRACO-164F-TCGGATAGACCTCCTTCTTC<br>NRACO-164R-CACCAATGGCAAGTACAAG	164	EF463066
		Reference gene		
8	Glyceraldehyde-3-phosphate dehydrogenase (GAPDH)	GAPDH-168F-TCACAGCCACTCAAAAGACTG<br>GAPDH-168R-TCGGAACACGGAAAGACATAC	168	AY821550.1

Table S2. Effect of SNP treatment on color parameters during banana fruit ripening.

Treatment	Color (Lightness#)				
	**Day 0**	**Day 5**	**Day 10**	**Day 15**	**Day 20**
Control	66.2 ± 3.28cd	68.54 ± 4.52bc	72.32 ± 5.46ab	69.52 ± 4.68bc	62.54 ± 2.82e
SNP 1 mM	64.52 ± 4.92de	66.62 ± 2.48cd	74.22 ± 3.16a	76.28 ± 4.88a	68.28 ± 4.22bc

Values are represented as Mean ± SE of six replicates. Significance was tested by Duncan Multiple Range Test at $p < 0.05$, and values with same superscript were found not significantly different from each other. #Lightness (L): A measure of the level of light or dark (ranges from black with a value of zero at the bottom of the axis to white with a value of 100 at the top of the axis).

Color of peel (Chroma)

Treatment	Color (Chroma$)				
	**Day 0**	**Day 5**	**Day 10**	**Day 15**	**Day 20**
Control	19.56 ± 2.42c	32.48 ± 5.82b	38.92 ± 4.16a	37.28 ± 4.28a	35.26 ± 3.84ab
SNP 1 mM	18.28 ± 1.92c	21.68 ± 3.46c	35.28 ± 3.64ab	38.22 ± 1.68a	36.52 ± 1.08a

Values are represented as Mean ± SE of six replicates. Significance was tested by Duncan Multiple Range Test at $p < 0.05$, and values with same superscript were found not significantly different from each other. $Chroma: A measure ranging from -150 to 150 increasing outward from the lightness axis; calculated as $(a2 + b2)^{0.5.}$

Color of peel (Hue)

Treatment	Color (Hue*)				
	**Day 0**	**Day 5**	**Day 10**	**Day 15**	**Day 20**
Control	−0.86 ± 0.1b	−0.96 ± 0.1b	−1.24 ± 0.3c	−1.62 ± 0.2d	1.92 ± 0.3a
SNP 1 mM	−0.81 ± 0.1b	−0.84 ± 0.1b	−0.94 ± 0.2b	−1.36 ± 0.2c	1.78 ± 0.2a

Values are represented as Mean ± SE of six replicates. Significance was tested by Duncan Multiple Range Test at $p < 0.05$, and values with same superscript were found not significantly different from each other. *Hue: A measure ranging from 010° to 360° as arranged on pages on the color coordinate axis; calculated as $\tan^{-1}$ (b/a). Hue angle values: 0°, 90°, 180° and 270° corresponds to red, yellow, green and blue colors respectively.
